# Machine learning decodes chemical features to identify novel agonists of a moth odorant receptor

**DOI:** 10.1038/s41598-020-58564-9

**Published:** 2020-02-03

**Authors:** Gabriela Caballero-Vidal, Cédric Bouysset, Hubert Grunig, Sébastien Fiorucci, Nicolas Montagné, Jérôme Golebiowski, Emmanuelle Jacquin-Joly

**Affiliations:** 1 INRAE, Sorbonne Université, CNRS, IRD, UPEC, Université Paris Diderot, Institute of Ecology and Environmental Sciences of Paris, Paris Versailles, France; 2 Institute of Chemistry of Nice, UMR CNRS 7272, Université Côte d’Azur, Nice, France; 3 Department of Brain and Cognitive Sciences, Daegu Gyeongbuk Institute of Science and Technology, Daegu, 711-873 South Korea

**Keywords:** Chemical ecology, Olfactory receptors

## Abstract

Odorant receptors expressed at the peripheral olfactory organs are key proteins for animal volatile sensing. Although they determine the odor space of a given species, their functional characterization is a long process and remains limited. To date, machine learning virtual screening has been used to predict new ligands for such receptors in both mammals and insects, using chemical features of known ligands. In insects, such approach is yet limited to Diptera, whereas insect odorant receptors are known to be highly divergent between orders. Here, we extend this strategy to a Lepidoptera receptor, SlitOR25, involved in the recognition of attractive odorants in the crop pest *Spodoptera littoralis* larvae. Virtual screening of 3 million molecules predicted 32 purchasable ones whose function has been systematically tested on SlitOR25, revealing 11 novel agonists with a success rate of 28%. Our results show that Support Vector Machine optimizes the discovery of novel agonists and expands the chemical space of a Lepidoptera OR. More, it opens up structure-function relationship analyses through a comparison of the agonist chemical structures. This proof-of-concept in a crop pest could ultimately enable the identification of OR agonists or antagonists, capable of modifying olfactory behaviors in a context of biocontrol.

## Introduction

Animals are exposed in their environment to a plethora of odorant molecules from a variety of chemical structures. Some of these molecules contain valuable information to carry out essential activities such as the identification of food sources, oviposition sites, mating partners, conspecifics and predators. Animals detect odorants via olfactory sensory neurons (OSNs) housed in dedicated olfactory organs, and the mechanisms underlying this detection have been particularly well studied in insects and mammals^1^. In insects, the primary olfactory organs consist of the antennae and the maxillary palps, which are covered by olfactory sensilla that house the OSNs^2^. In mammals, OSNs are mainly localized within the olfactory epithelium of the nasal cavity. In both insects and mammals, large multigenic families of odorant receptor proteins (ORs) mediate odorant recognition, each OSN expressing a single receptor (plus Orco in insects, see below) that controls its detection spectrum. These ORs are seven-transmembrane (TM) domain receptors^3–5^, yet mammalian and insect ORs belong to distinct unrelated families^6^. Mammalian ORs are members of the class A rhodopsin-like G protein–coupled receptors (GPCR)^7^, whereas insect OR membrane topology is opposite to that of GPCRs, with a cytoplasmic N-terminus and an extracellular C-terminus^8^. Furthermore, insect ORs form heteromers with a well conserved coreceptor named Orco^8–10^, and these heteromers are gated directly by chemical stimuli^11^.

Understanding how the OR repertoire of an animal contributes to odor sensing and adaptation to a specific environment relies on the capacity to identify natural ligands of these ORs, a process called deorphanization. Yet, the ligands of several mammalian and insect ORs have been identified using different expression systems^12–19^. However, the number of chemicals used to stimulate the ORs is limited due to practical handling and duration of the experimentation. Consequently, potential stimuli that are tested on ORs of a given species are generally only a small portion of the vast array of ecologically relevant odorants. In insects, such sets of potential stimuli consisted of up to 100 molecules used to challenge *Drosophila melanogaster*^19^ (even up to 500 in one study but with only one replicate^20^) and *Anopheles gambiae* ORs^16,17^, but only fifty have been used to stimulate the ORs of a moth, *Spodoptera littoralis*^18^. Given that the potential odor space for an animal is almost unlimited, it is likely that the main ligand(s) of some deorphanized ORs still remains unidentified. The problem of selecting the candidate molecules to be tested becomes even more critical when trying to identify agonists or antagonists of particular ORs that are not natural ligands but could have an impact on the behavior of pest and disease vector insects^21^.

Several recent studies revealed that the application of machine learning in the context of virtual screening opens up the possibility to enlarge animal odor spaces. Machine learning based on odorant chemical descriptors allowed predicting receptor–odorant interactions in both insects^22–25^ and mammals^26^, although their ORs do not belong to the same protein families. Notably, quantitative structure-activity relationship (QSAR) is an *in silico* ligand-based method used to predict biological activity of untested chemicals, based on chemical features shared by active molecules^27^. In *D. melanogaster*, virtual screening of more than 240,000 chemical structures identified a large array of novel OR activators and inhibitors^25^. An *in silico* screening of 0.5 million compounds identified agonists or antagonists targeting the mosquito CO_2_ receptor, leading to the discovery of new attractants and repellents for those harmful disease vectors^24^. More recently, antagonists for the insect coreceptor Orco have been identified by screening a library of 1280 odorant molecules^28^. In mammals, a more modest virtual screening of 258 chemicals anyhow identified new agonists of four human ORs^26^. Although efficient, this approach requires prior knowledge on the response spectrum of a given OR and its application has thus been restricted to model species with cumulative odorant-receptor functional data.

We have recently deorphanized a large array of ORs in the noctuid moth *Spodoptera littoralis* through heterologous expression in *Drosophila* OSNs^18^. This offers an unprecedented opportunity to test such a computational approach in a non-dipteran insect. *Spodoptera littoralis* is a polyphagous moth^29^ present in Africa, the Middle East and Southern Europe^30^. At the larval stage, *S. littoralis* is responsible for extensive damage in a large number of crops of economic importance^29^. Establishing machine learning virtual screening efficiency in such an herbivorous pest species will open new routes for the identification of possible agonists and antagonists to be used in biocontrol strategies. In addition, screening structurally related molecules can bring crucial information to determine structure-function relationships. Here, we focused on *S. littoralis* OR25 (SlitOR25), an odorant receptor that is particularly suitable for this approach. Over a panel of 52 volatile organic compounds, SlitOR25 is strongly activated by nine agonists and moderately activated by four^18^. Also, it is expressed at both larval and adult stages and its activation has been correlated with caterpillar attraction^31^. Based on properties of the previously identified SlitOR25 ligands, we carried out an *in silico* screening of a chemical space of more than three million chemicals, leading to the prediction of 90 potential agonists, of which 32 were commercially available. The activity of these 32 compounds was further functionally tested on SlitOR25 expressed in *Drosophila* OSNs. We revealed enrichment of SlitOR25 agonists, with a hit rate of 28%. With the current lack of any OR structure - apart that of Orco^32^-, this machine-learning protocol based on chemical molecular descriptors thus represents an efficient tool for addressing ligand structure-function relationship in addition to identifying novel unexpected ligands for moth ORs, extending their odor space outside the presupposed relevant odorants.

## Results and Discussion

### *In silico* prediction of SlitOR25 agonists

**Figure 1:**
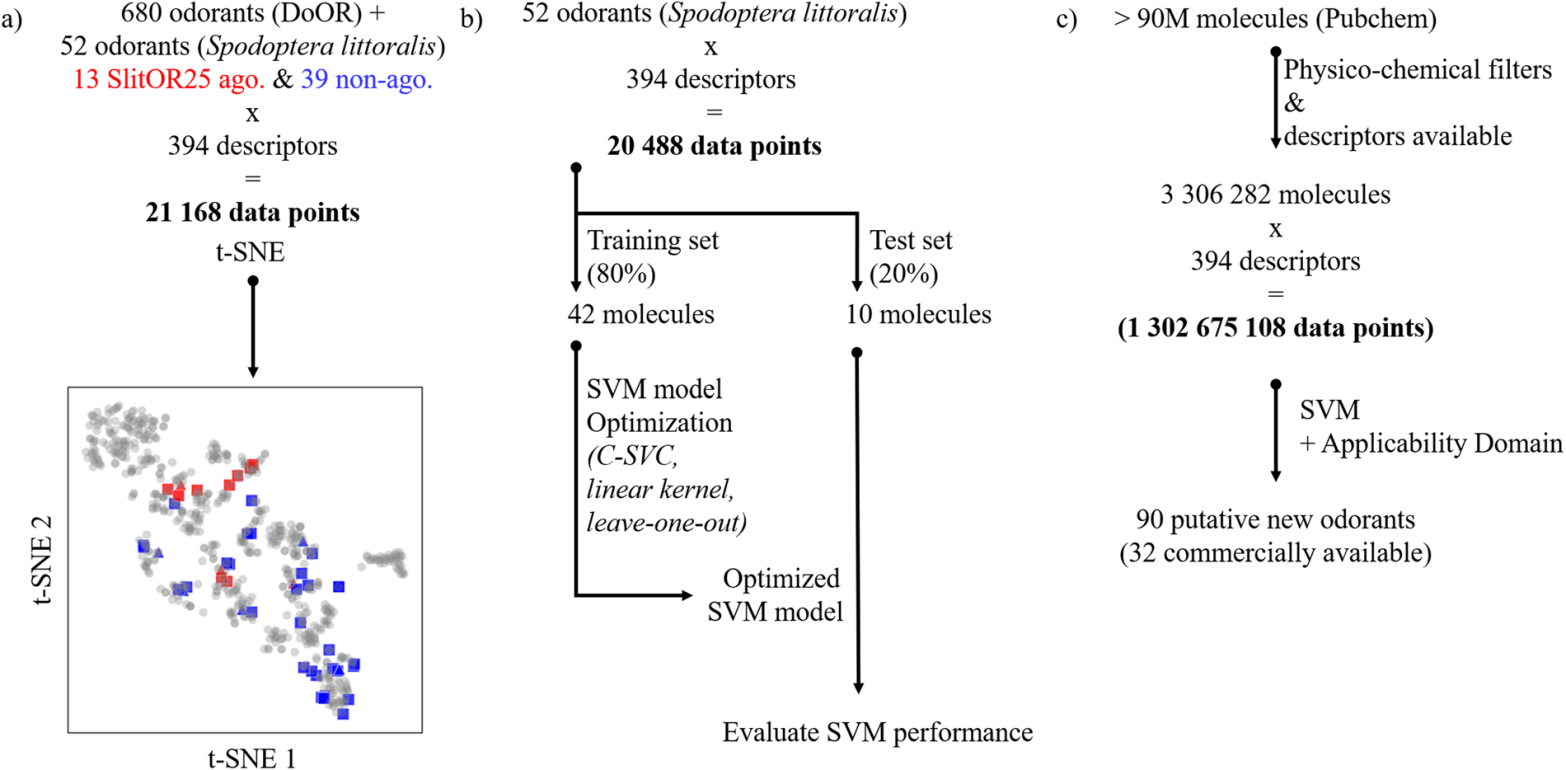
Analysis of insect odorant molecular space and protocol used for *Spodoptera littoralis* OR25 (SlitOR25) virtual screening. **(a)** Visualization of SlitOR25 and *Drosophila melanogaster* olfactory chemical spaces based on a t-distributed stochastic neighbor embedding (t-SNE) dimensionality reduction method. The agonists (ago) and non-agonists (non-ago) of SlitOR25 are shown in red and blue, respectively, and agonists of *D. melanogaster* are shown in gray. Chemicals of the training set are shown in squares while those of the test set are shown as triangles **(b)** Workflow of the Support Vector Machine (SVM) model based on an 80%/20% split of the initial database. Forty-two molecules constituting the training set were used to find optimized SVM parameters while 10 molecules were kept for a blind evaluation by the optimized SVM (Supplementary Table S1). C-SVC: C-Support Vector Classification. **(c)** Virtual screening of more than three million molecules extracted from the PubChem database resulted in 90 agonist candidates.

First, the published SlitOR25 chemical space^18^ was analyzed through calculation of its known ligand chemical descriptors and projection on the *Drosophila melanogaster* Database of Odorant Responses (DoOR v2.0)^33^, considered as prototypical. Figure 1a simplifies this chemical space using a t-distributed stochastic neighbor embedding (t-SNE) algorithm in two dimensions. Agonists were split into two distinct clusters, suggesting that a machine learning model (Fig. 1b) should be able to identify rules to separate them from non-agonists (see Supplementary Table S1 for a list of the considered molecules). Then, the external dataset to be screened was obtained by filtering ~90 million molecules from the PubChem database as described in the method section. More than three million molecules corresponding to organic potentially volatile molecules were extracted and were evaluated by the optimized Support Vector Machine (SVM). After an additional filter associated with the applicability domain obtained by a similarity search with the known agonists, 90 molecules were predicted as agonists (Fig. 1c and Supplementary Table S2). The performance of the SVM is resumed in Table 1 and Supplementary Table S3.

**Table 1:** Five-fold random split Support Vector Machine performance metrics. %CC: percentage of instances correctly classified, MCC: Matthews Correlation Coefficient.

Dataset	%CC	Precision	Recall	MCC
Training	0.90 ± 0.03	0.77 ± 0.05	0.84 ± 0.08	0.77 ± 0.07
Test	0.92 ± 0.06	0.88 ± 0.16	0.91 ± 0.12	0.83 ± 0.12

### Effect of predicted agonists on SlitOR25 activity

**Figure 2:**
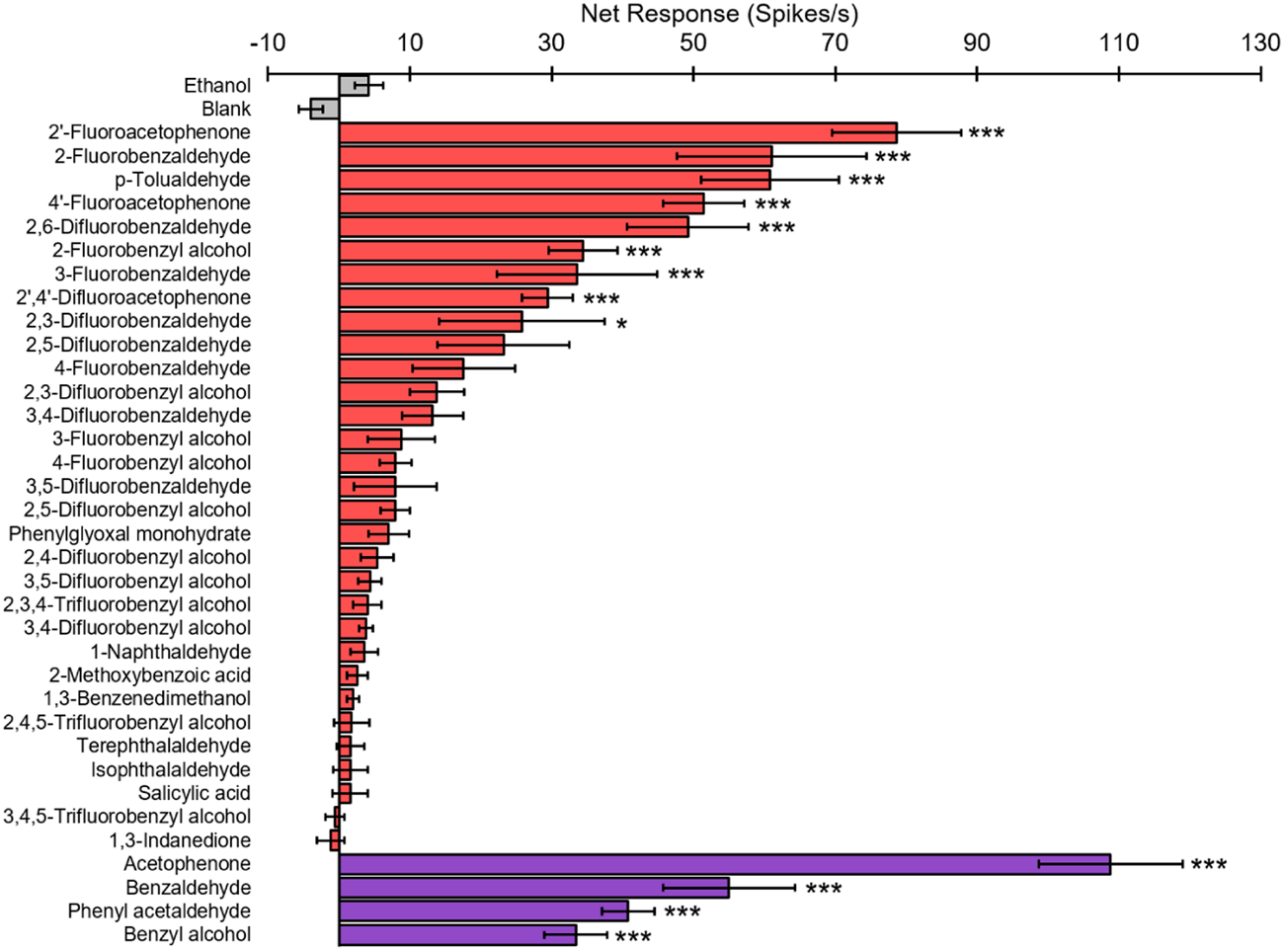
Response of *Drosophila* ab3A OSNs expressing SlitOR25 to 32 candidate ligands predicted via ligand-based QSAR approach. Responses are presented ± s.e.m. Grey bars: controls (ethanol solvent, blank). Red bars: predicted compounds tested in SSR at high doses (10^−2^, ethanol dilution). Purple bars: known SlitOR25 ligands used as positive controls^18^ (10^−2^, ethanol dilution). Asterisks indicate statistically significant differences between responses to the odorant and to the solvent (Kruskal–Wallis test followed by a Dunnett multiple comparison test, *p < 0.05, ***p < 0.001, n = 10).

Among the predicted novel agonists of SlitOR25, 32 molecules were commercially available at high purity (Table 2). These molecules were mainly fluorinated derivatives of known ligands (acetophenone, benzyl alcohol, benzaldehyde). To verify whether these were indeed agonists of SlitOR25, we performed single-sensillum recordings on *D. melanogaster* flies expressing SlitOR25 in ab3A OSNs instead of the endogenous receptor OR22a, a heterologous expression system known as the “empty neuron”^34^. A first screen with a high concentration of the 32 candidate agonists (10^−2^ dilution) revealed that nine of them elicited a significant response (p < 0.05, Fig. 2), representing a 28% success rate. For comparison, 30% of 138 *in silico* predicted odorants activated the mosquito CO_2_ receptor in a first round^24^. Machine learning models based on ligand topology predicted 138 antagonists for mosquito Orco, out of which 45 were active (32%)^28^. In this last study, it has to be noticed that 58 active antagonists were used to feed the machine learning, a number that is much higher than the 13 ligands we used. In *Drosophila*, another study revealed that the success rate of an optimized QSAR greatly depends on the receptor (varying from 27% to 71%)^25^ and that lowest rates were obtained for ORs tuned to aromatics (around 30%). Here, we add new evidences that machine learning is of great help to discover novel ligands for Lepidoptera ORs.

**Table 2:** Predicted agonists (this study) and known ligands^18^ (in bold) tested on SlitOR25.

Compounds	CAS	Provider	Purity
1-Naphthaldehyde	66-77-3	Alfa Aesar	97%
2′-Fluoroacetophenone	445-27-2	Alfa Aesar	97%
Phenylglyoxal monohydrate	1074-12-0	Acros organics	97%
Terephthalaldehyde	623-27-8	Alfa Aesar	98%
Isophthalaldehyde	626-19-7	Alfa Aesar	98%
1,3-benzenedimethanol	626-18-6	Alfa Aesar	98%
2-Fluorobenzaldehyde	446-52-6	Alfa Aesar	97%
2-Fluorobenzyl alcohol	446-51-5	Alfa Aesar	98%
4-Fluorobenzaldehyde	459-57-4	Alfa Aesar	98%
4-Fluorobenzyl alcohol	459-56-3	Alfa Aesar	97%
3,4-Difluorobenzaldehyde	34036-07-2	Alfa Aesar	98%
3,4-Difluorobenzyl alcohol	85118-05-4	Alfa Aesar	99%
2,3,4-Trifluorobenzyl alcohol	144284-24-2	Alfa Aesar	97%
Salicylic acid	69-72-7	VWR chemicals	98%
3-Fluorobenzyl alcohol	456-47-3	Alfa Aesar	98%
3- Fluorobenzaldehyde	456-48-4	Alfa Aesar	97%
2,5-Difluorobenzaldehyde	2646-90-4	Alfa Aesar	98%
2,6-Difluorobenzaldehyde	437-81-0	Alfa Aesar	97%
3,5-Difluorobenzyl alcohol	79538-20- 8	Alfa Aesar	97%
3,5-Difluorobenzaldehyde	32085-88-4	Alfa Aesar	97%
2,4-Difluorobenzyl alcohol	56456-47-4	Alfa Aesar	98%
2,4-Difluorobenzaldehyde	1550-35-2	Alfa Aesar	98%
2,3-Difluorobenzaldehyde	2646-91-5	Alfa Aesar	98%
3,4,5-Trifluorobenzyl alcohol	220227-37-2	Alfa Aesar	97%
2,4,5-Trifluorobenzyl alcohol	144284-25-3	Alfa Aesar	98%
1,3-Indanedione	606-23-5	Alfa Aesar	97%
p-tolualdehyde	104-87-0	Alfa Aesar	98%
4′-Fluoroacetophenone	403-42-9	Alfa Aesar	99%
2′,4′-Difluoroacetophenone	364-83-0	Alfa Aesar	98%
2-Methoxybenzoic acid	579-75-9	Alfa Aesar	98%
2,3-Difluorobenzyl alcohol	75853-18- 8	Alfa Aesar	97%
2,5-Difluorobenzyl alcohol	75853-20- 2	Alfa Aesar	98%
**Benzaldehyde**	100-52-7	Sigma-Aldrich	99,5%
**Z-3-hexenol**	928-96-1	Sigma-Aldrich	98%
**Methyl salicylate**	119-36-8	Sigma-Aldrich	99%
**2-phenyl acetaldehyde**	122-78-1	Sigma-Aldrich	98%
**Benzyl methyl ether**	538-86-3	Sigma-Aldrich	98%
**Methyl benzoate**	93-58-3	Acros organics	97%
**Benzyl alcohol**	100-51-6	Sigma-Aldrich	99%
**Acetophenone**	98-86-2	Acros organics	99%
**E-2-hexenol**	928-95-0	Sigma-Aldrich	96%
**E-2-hexenal**	6728-26-3	Sigma-Aldrich	98%
**1-hexanol**	111-27-3	Sigma-Aldrich	98%
**1-heptanol**	111-70-6	Sigma-Aldrich	99%

Looking in detail at the new ligands identified for SlitOR25, none presented a reverse agonist activity (reduction of spontaneous activity), whereas this has been observed for 13% of predicted ligands for *D. melanogaster* ORs when tested on OSNs^25^. This is likely attributed to the nature of the screened receptor, where reverse agonists would be part of a far-removed chemical space compared to agonists. However, with the current lack of any structure of an insect OR (apart that of Orco)^32^, providing a mechanistic view on the way agonists work is extremely difficult.

### Dose-response analyses

**Figure 3:**
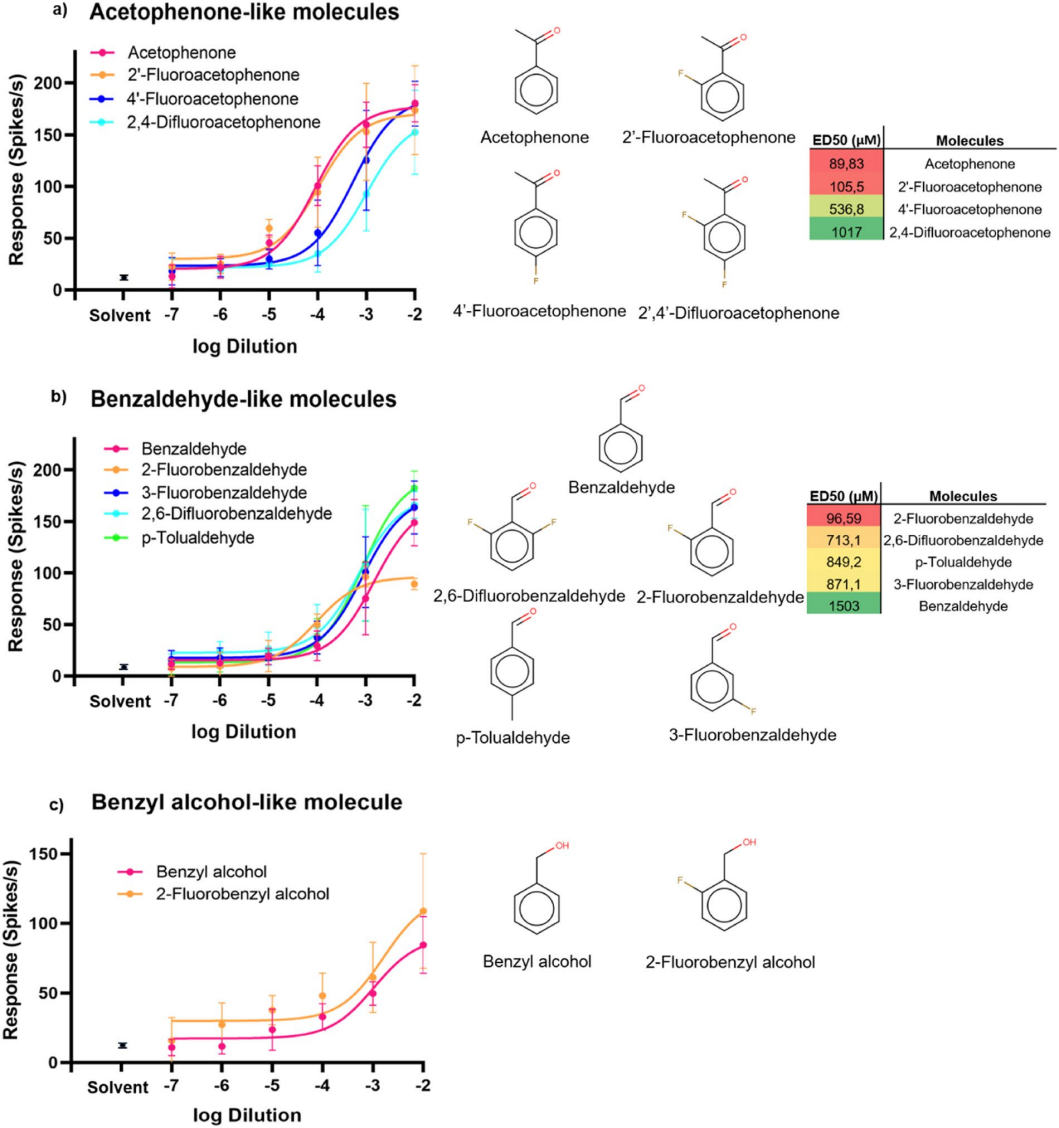
Dose-response activities (measured via SSR), structures and ED50 values of newly identified and three previously identified ligands on SlitOR25 expressed in *Drosophila* ab3A OSNs. SSR responses are presented ± s.e.m. Only molecules with a significant activity in the screening tests (p < 0.05, 10^−2^ dilution, Fig. 2) were tested in dose-response using dilutions from 10^−7^ to 10^−2^. (**a**) Molecules structurally related to the known ligand acetophenone: 2′-fluoroacetophenone, 4′-fluoroacetophenone, 2′,4′-difluoroacetophenone. (**b**) Molecules related to the ligand benzaldehyde: 2-fluorobenzaldehyde, 3-fluorobenzaldehyde, 2,6-difluorobenzaldehyde, p-tolualdehyde. (**c**) Molecule related to the ligand benzyl alcohol: 2-fluorobenzyl alcohol.

To compare the responses evoked on SlitOR25 by the nine newly identified agonists to those evoked by the previously known natural ligands, we conducted dose-response SSR experiments, using dilutions ranging from 10^−7^ to 10^−2^, and effective doses 50 (ED50s) were calculated. Statistical analyses for the responses of all molecules tested in dose-response are detailed in Table 3. For predicted molecules structurally related to the ligand acetophenone (Fig. 3a), statistically significant responses (p < 0.05) were observed for all tested molecules from 10^−6^ dilution. For the newly predicted ligands structurally related to benzaldehyde (Fig. 3b), detection thresholds varied from 10^−7^ (2,6-difluorobenzaldehyde) to 10^−4^ (2-fluorobenzaldehyde) dilution, whereas that of benzaldehyde was 10^−6^. The predicted agonist 2-fluorobenzyl alcohol exhibited a higher activation threshold than the structurally related-known ligand benzyl alcohol (Fig. 3c). Our results demonstrated that machine learning was very efficient in identifying new strong ligands for SlitOR25.

**Table 3:** Statistics for the responses of SlitOR25 to known (acetophenone, benzyl alcohol and benzaldehyde) and new ligands at different doses. Solvent: ethanol.

Tested molecules	Dilutions					
	10^−7^	10^−6^	10^−5^	10^−4^	10^−3^	10^−2^
Acetophenone	NS	***	***	***	***	***
Benzyl alcohol	NS	NS	NS	***	***	***
Benzaldehyde	NS	***	***	***	***	***
2′-Fluoroacetophenone	NS	***	***	***	***	***
2-Fluorobenzaldehyde	NS	NS	NS	***	***	***
2-Fluorobenzyl alcohol	NS	NS	***	***	***	***
3-Fluorobenzaldehyde	NS	*	***	***	***	***
2,6-Difluorobenzaldehyde	***	***	***	***	***	***
p-Tolualdehyde	NS	NS	***	***	***	***
4′-Fluoroacetophenone	NS	***	***	***	***	***
2′,4′-Difluoroacetophenone	***	***	***	***	***	***

Asterisks indicate statistically significant differences between responses to the odorant and to solvent (Kruskal–Wallis test followed by a Dunnett multiple comparison test, *p < 0.05, ***p < 0.001, n = 5).

Independent of the pharmacophore approach and by visually inspecting the structures, the presence of a Fluor atom at the ortho position in the ring (position 2) maintains the agonist behaviour for the three chemical families (aldehydes, ketones, alcohol). Multiple fluorinations had either a weak beneficial effect on benzaldehyde derivatives or decreased or abolished the response in other series (Supplementary Fig. S1).

The predicted molecules we functionally tested present strongly intertwined chemical spaces. The functional assays we conducted revealed that some were strong agonists and other were non-agonists (Supplementary Fig. S1), allowing us to tentatively recapitulate the features required for being an agonist through a pharmacophore approach (Supplementary Fig. S2). However, the model was not able to discriminate agonist from non-agonists based on the position of the Fluor atom on the aromatic cycle.

Alternatively, a statistical analysis of the descriptors able to discriminate between agonists and non-agonists revealed 105 descriptors out of the 394 processed initially. These descriptors can either be constitutional, topologic, or electronic. They are challenging to interpret but could serve as a basis for a further screening protocol.

## Conclusion

### Machine learning widens the chemical space of a moth odorant receptor

In this study, we have used machine learning to predict novel agonists for SlitOR25, a broadly tuned receptor in the Lepidoptera *S. littoralis*. A Support Vector Machine was fed with 52 ligands for which the activity was already reported. After optimization, a database of more than 90 million chemicals was filtered and screened. Out of the three million of potentially useful molecules, 90 were predicted as agonists, of which 32 were commercially available. *In vivo* functional assays and dose-response analyses on these latter assessed nine novel molecules as moderate or strong agonists for the receptor.

Modeling has already been shown to provide accurate information and facilitate the selection of active molecules on odorant receptors. In insects, it has been applied only in two Diptera models, the fruit fly and the mosquito^24,25,28^. In this study, we reveal that a conventional machine learning approach is efficient for the identification of novel agonists for a moth receptor, whose amino acid sequence is unrelated to that of Diptera ORs.

It has to be noticed that none of the novel agonists discovered here has been previously described in the literature to be active on moth ORs and most are not described as plant emitted volatiles. Although they may not be encountered by insects in the wild, we have anyhow extended the chemical space of *S. littoralis* and the cumulated results open up ligand structure-function relationship analyses. More importantly, closed-loop machine learning is now possible, where the new highly potent agonists discovered here could be used to train new models, further improving predictions in alternative and far removed chemical spaces.

## Methods

### Reagents

Reagents were purchased from various vendors (Table 2) at the highest available purity (ranging from 96 to 99% depending on the molecules) and were dissolved in ethanol (96% purity, Carlo Erba reagents).

### Quantitative structure activity relationship

#### Softwares

Knime v3.2.1 was used to build the workflow, chemical descriptors were computed with Dragon v6.0.40 and the LibSVM v2.89 was used for the machine learning protocol^35^.

#### Training and test sets

The initial database of 52 volatiles (Supplementary Table S1) was obtained from^18^. The previously identified strong agonists of SlitOR25 were benzenoids (acetophenone, benzyl alcohol, benzaldehyde, phenyl acetaldehyde, 1-indanone) and short aliphatic alcohols and aldehydes (1-hexanol, 1-heptanol, (Z)3-hexenol, (E)2-hexenal)^18^, which are compounds emitted mainly by flowers and leaves^36^. The receptor also responds to four other molecules (methyl salicylate, methyl benzoate, benzyl methyl ether, (E)2-hexenol), with weaker but still significant responses. The SlitOR25 database thus contains 13 agonists and 39 non-agonists. It was randomly split into a training set of 42 molecules and a test set of 10 molecules. Molecules of the training set were considered for the optimization of the model. Those of the test set were not used to build the model but to assess its performance.

#### External test set

3 306 388 molecules out of 90 million were extracted from the Pubchem database^37^ according to the following physico-chemical properties obtained directly on the website: each molecule has to contain a combination of C, H, O, N, F, S, or Cl elements with less than 20 heavy atoms, a molecular weight lower than 200 g.mol^−1^, and a LogP in the range [0, 5].

#### Chemical space analysis

The Database of Odorant Response (DoOR v2.0)^33^ was used to analyze the *S. littoralis* chemical space that has been used to train the machine learning model. Excluding salts from the analysis, DoOR contains 680 odorants that have been experimentally tested on *D. melanogaster*. The t-SNE dimensionality reduction method was used to evaluate how our database of 52 ligands span a typical insect chemical space.

#### Molecular descriptors

For each dataset of the QSAR model (training, test and external sets) 4885 descriptors were computed using the *Dragon* software (version 6.0.40) based on 3D sdf files obtained directly from Pubchem. Constant or near-constant (variance lower than 0.005) descriptors were excluded from the database as well as descriptors with at least one missing value. Each descriptor of the final matrix was normalized using a min-max protocol (range [0,1]) before the split between training and test sets. Note that a normalization before or after the split did not affect the nature of the predicted agonists. Redundant descriptors were removed (absolute pair correlation greater than or equal to 0.95). The final SVM matrix contained 394 molecular descriptors. It was used for the t-SNE visualization of the database containing both the *S. littoralis* and *D. melanogaster* chemical spaces (see supplementary Fig. S3 for details on t-SNE). The descriptors were computed on a machine with an intel Xeon with 32 GB of memory.

#### Setting up the QSAR model

Various numerical models, such as Random Forest or Perceptron (data not shown), were tested prior optimizing the chosen supervised machine learning method, the Support Vector Machine (SVM). A brute force optimization was applied to assess the exhaustive parameter value combination. The C-SVC (C-Support Vector Classification) model with a linear kernel was finally used.

The C-SVC parameters were optimized in a two-step process. First a 5-fold-random split was performed with a cost ranging from 1 to 10 with a step of 1. Epsilon varied between 0.0001 and 0.1 with a step of 0.01. The model’s accuracy remained identical for values in this range. Second, a more precise 5-fold-random split sampling was performed, with a cost between 0.5 and 1.5 using a step of 0.1, and epsilon between 0.001 and 0.01 with a step of 0.001. Again, the accuracy was identical to that obtained with default settings (accuracy 0.9 ± 0.09).

The optimized SVM parameters were accordingly set as follows: cost = 1.0, epsilon 0.001. The leave-one-out cross validation method was used. Each of the 13 agonists was given a score of 1 and the non-agonists were given a score of 0.

#### Applicability domain

A Tanimoto score that measures the similarity between compounds and varies between 0 and 1 (whereby a value closer to 1 indicates greater similarity) was calculated from Pubchem molecular fingerprints (881 Pubchem molecular descriptors obtained from the CDK module of Knime). The use of Pubchem fingerprints has already been shown to correctly capture biological activities^38^. Putative new odorants which had a Tanimoto index higher than 0.92 with respect to the Training set were considered belonging to the applicability domain. In our case, this corresponds to 90 molecules.

### Single-sensillum recordings of *Drosophila* olfactory sensory neurons

Flies were reared on standard cornmeal-yeast-agar medium (25 °C, 12 h light: 12 h dark cycle). SlitOR25-expressing flies were obtained by crossing the line *w;Δhalo/CyO;UAS-SlitOr25*^18^ with the line *w; Δhalo/CyO;Or22a-Gal4*^34^. Single-sensillum recordings were conducted as previously described^18^. Briefly, a 2- to 8-day-old fly was placed on a microscope glass slide under a constant 1.5 L.min^−1^ flux of charcoal-filtered and humidified air delivered through a glass tube of a 7 mm diameter, and observed with a light microscope (BX51WI, Olympus, Tokyo, Japan) equipped with a 100X magnification objective. Action potentials from ab3A OSNs were recorded using electrolytically sharpened tungsten electrodes (TW5-6, Science Products, Hofheim, Germany).

Stimulus cartridges were built by placing a 1 cm^2^ filter paper in a Pasteur pipette and loading 10 µl of the odorant solution onto the paper (10^−2^ dilution in ethanol), or 10 µL of ethanol as control. Evaporation time before using the cartridge was 10 minutes. Odorant stimulations were performed by inserting the tip of the pipette into a hole in the glass tube and generating a 500 ms air pulse (0.6 L.min^−1^). The responses of ab3A OSNs were calculated as in^39^ by subtracting the spontaneous firing rate (in spikes.s^−1^) from the firing rate during the odorant stimulation.

The absence of the endogenous receptor OR22a in ab3A OSNs was verified using ethyl hexanoate (a strong ligand of OR22a) as a stimulus. Then, the SlitOR25 response spectrum was established using the panel of 32 predicted agonists (Table 2) and four already known ligands as controls. The stimulus cartridges were used at most twice per fly (and maximum eight times in total). The entire panel of molecules was tested ten times on ten different flies expressing SlitOR25. Odorants were considered as active if the response was statistically different from the response elicited by the solvent alone (Kruskal–Wallis test, followed by a Dunnett multiple comparison test, p < 0.05).

For molecules that yielded a statistically significant response, dose-response experiments were conducted with odorant dilutions ranging from 10^−2^ down to 10^−7^. Each dilution was tested in five different flies expressing SlitOR25. ED50 were calculated (except for benzyl alcohol and 2-fluorobenzyl alcohol) using GraphPad PRISM V.8.1.2 software.

### SlitOR25 pharmacophore hypothesis

For the generation of the SlitOR25 pharmacophore, we considered a dataset of eleven odorants that are active on SlitOR25, as well as fourteen inactive compounds. All these molecules are derivatives of acetophenone described in this work. The pharmacophore was generated with up to four features, chosen between H-bond donors/acceptors, hydrophobic sites, and aromatic rings. Even considering several conformations for each molecule, the pharmacophore hypotheses generated by the software CATALYST (version 4.9.1, Accelrys Inc., San Diego, CA, August 2004) were identical, comprised of an aromatic ring and a H-bond acceptor. The addition of exclusion volumes did not improve the model and was thus discarded.

## Supplementary information

Supplementary Information.
