# Supplementary material for: Machine learning decodes chemical features to identify novel agonists of a moth odorant receptor
Source: Sci Rep. 2020 Feb 3;10:1655. doi: 10.1038/s41598-020-58564-9 (PMC6997167; doi:10.1038/s41598-020-58564-9)
Supplement: Supplementary file 1 — Supplementary Information. [file 41598_2020_58564_MOESM1_ESM.docx]

**Supplementary Information**

**Machine learning decodes chemical features to identify novel agonists of a moth odorant receptor**

Gabriela Caballero-Vidal^1,+^, Cédric Bouysset^2,+^, Hubert Grunig^2^, Sébastien Fiorucci^2^, Nicolas Montagné^1*^, Jérôme Golebiowski^2,3*^, and Emmanuelle Jacquin-Joly^1*^

^1^INRA, Sorbonne Université, CNRS, IRD, UPEC, Université Paris Diderot, Institute of Ecology and Environmental Sciences of Paris, Paris and Versailles, France

^2^Institute of Chemistry of Nice, UMR CNRS 7272, Université Côte d’Azur, Nice, France

^3^Department of Brain and Cognitive Sciences, Daegu Gyeongbuk Institute of Science and Technology, Daegu 711-873, South Korea

^+^both authors contributed equally to the work

* corresponding authors:

[emmanuelle.joly@inra.fr](mailto:emmanuelle.joly@inra.fr)

[jerome.golebiowski@unice.fr](mailto:jerome.golebiowski@unice.fr)

[nicolas.montagne@sorbonne-universite.fr](mailto:nicolas.montagne@sorbonne-universite.fr)

**Supplementary** **Table S1.** Database of molecules used to train the machine learning model. Agonists marked with an * were not considered as strong agonists in the work by de Fouchier et al 2017^18^, but the receptor response was still significantly different from solvent. They were thus included in our agonist list.

| **NAME** | **CAS** | **Classification** | **Training / Test set** |
| --- | --- | --- | --- |
| benzaldehyde | 100-52-7 | agonist | training set |
| phenylacetaldehyde | 122-78-1 | agonist | training set |
| (E)2-hexenal | 6728-26-3 | agonist | training set |
| (E)2-hexenol | 928-95-0 | agonist* | training set |
| (Z)3-hexenol | 928-96-1 | agonist | training set |
| 1-hexanol | 111-27-3 | agonist | training set |
| 1-heptanol | 111-70-6 | agonist | test set |
| benzyl alcohol | 100-51-6 | agonist | test set |
| acetophenone | 98-86-2 | agonist | training set |
| 1-indanone | 83-33-0 | agonist | training set |
| methyl salicylate | 119-36-8 | agonist* | training set |
| methyl benzoate | 93-58-3 | agonist* | training set |
| benzyl methyl ether | 538-86-3 | agonist* | test set |
| 1-octanol | 111-87-5 | non agonist | training set |
| (Z)9-14: OH | 35153-15-2 | non agonist | training set |
| (Z)7-12:OAc | 14959-86-5 | non agonist | test set |
| (Z,E)-9,12-14:OAc | 30507-70-1 | non agonist | training set |
| (Z)-jasmone | 488-10-8 | non agonist | training set |
| α-copaene | 3856-25-5 | non agonist | training set |
| nonanal | 124-19-6 | non agonist | training set |
| sulcatone | 110-93-0 | non agonist | test set |
| α-humulene | 6753-98-6 | non agonist | training set |
| (E)11-14:OAc | 33189-72-9 | non agonist | training set |
| TMTT | 62235-06-7 | non agonist | training set |
| (Z)11-14:OAc | 20711-10-8 | non agonist | training set |
| decanal | 112-31-2 | non agonist | training set |
| (Z)9-14:OAc | 16725-53-4 | non agonist | test set |
| methyl jasmonate | 39924-52-2 | non agonist | training set |
| (E,E)-α-farnesene | 502-61-4 | non agonist | training set |
| (±)-linalool | 78-70-6 | non agonist | training set |
| (±)-phytol | 7541-49-3 | non agonist | training set |
| carvacrol | 499-75-2 | non agonist | training set |
| eugenol | 97-53-0 | non agonist | test set |
| β-myrcene | 123-35-3 | non agonist | training set |
| (±)-nerolidol | 7212-44-4 | non agonist | training set |
| hexane | 110-54-3 | non agonist | training set |
| β-caryophyllene | 87-44-5 | non agonist | test set |
| DMNT | 19945-61-0 | non agonist | training set |
| 1-nonanol | 143-08-8 | non agonist | training set |
| EDD | 3025-30-7 | non agonist | training set |
| 3-carene | 13466-78-9 | non agonist | test set |
| 14:OAc | 638-59-5 | non agonist | training set |
| indole | 120-72-9 | non agonist | training set |
| (Z,E)-9,11-14:OAc | 50767-79-8 | non agonist | training set |
| geraniol | 106-24-1 | non agonist | training set |
| (Z)3-hexenyl acetate | 3681-71-8 | non agonist | training set |
| β-pinene | 127-91-3 | non agonist | training set |
| (E)-ocimene | 3779-61-1 | non agonist | test set |
| α-pinene | 80-56-8 | non agonist | training set |
| estragole | 140-67-0 | non agonist | training set |
| thymol | 89-83-8 | non agonist | training set |
| (E,E)-farnesol | 106-28-5 | non agonist | training set |

**Supplementary** **Table S2.** Panel of 90 predicted agonist molecules for SlitOR25.

| **NAME** | **CID** | **CAS (if available)** |
| --- | --- | --- |
| Salicylic acid | 338 | 69-72-7 |
| P-Tolualdehyde | 7725 | 104-87-0 |
| 4'-Fluoroacetophenone | 9828 | 403-42-9 |
| 2-Fluoroacetophenone | 9947 | 450-95-3 |
| 2-methoxybenzoic acid | 11370 | 579-75-9 |
| 1,3-Indanedione | 11815 | 606-23-5 |
| terephthalaldehyde | 12173 | 623-27-8 |
| Pent-2-enal | 12993 | 764-39-6 |
| 2-oxo-2-phenylacetaldehyde | 14090 | 1074-12-0 |
| 2-Penten-1-ol | 15306 | 1576-95-0 |
| Isophthalaldehyde | 34777 | 626-19-7 |
| 2',4'-Difluoroacetophenone | 67770 | 364-83-0 |
| 2-Fluorobenzyl alcohol | 67969 | 446-51-5 |
| 2-Fluorobenzaldehyde | 67970 | 446-52-6 |
| 3-Fluorobenzyl alcohol | 68008 | 456-47-3 |
| 3-Fluorobenzaldehyde | 68009 | 456-48-4 |
| 4-Fluorobenzyl alcohol | 68022 | 459-56-3 |
| 4-Fluorobenzaldehyde | 68023 | 459-57-4 |
| 1,3-Benzenedimethanol | 69374 | 626-18-6 |
| 2,4-Difluorobenzaldehyde | 73770 | 1550-35-2 |
| (2,6-difluorophenyl)methanol | 87921 | 19064-18-7 |
| 2,4-Difluorobenzyl alcohol | 91867 | 56456-47-4 |
| 2'-Fluoroacetophenone | 96744 | 445-27-2 |
| 3H-indene-1,2-dione | 123358 | 16214-27-0 |
| 2,6-difluorobenzaldehyde | 136284 | 437-81-0 |
| 2,5-Difluorobenzaldehyde | 137663 | 2646-90-4 |
| 2,3-Difluorobenzaldehyde | 137664 | 2646-91-5 |
| Benzocyclobutenone | 137953 | 3469-06-5 |
| Hydroperoxy(phenyl)methanol | 286896 |  |
| 2,3-Difluorobenzyl alcohol | 447153 | 75853-18-8 |
| 2,5-Difluorobenzyl alcohol | 522599 | 75853-20-2 |
| 3,5-Difluorobenzyl alcohol | 522721 | 79538-20-8 |
| 3,4-Difluorobenzyl alcohol | 522833 | 85118-05-4 |
| hex-3-ene-1,6-diol | 549321 | 67077-43-4 |
| 3,4-Difluorobenzaldehyde | 588088 | 34036-07-2 |
| 3,5-Difluorobenzaldehyde | 588160 | 32085-88-4 |
| 4H-naphthalen-1-one | 2754230 | 19369-49-4 |
| (2,3,4-trifluorophenyl)methanol | 2777027 | 144284-24-2 |
| 2,4,5-Trifluorobenzyl alcohol | 2777035 | 144284-25-3 |
| 3,4,5-Trifluorobenzyl alcohol | 2777040 | 220227-37-2 |
| 2-phenylmalonaldehyde | 3672296 | 26591-66-2 |
| 2-fluorohexan-1-ol | 10441694 | 1786-48-7 |
| 2-Fluoroindan-1-one | 11029998 | 700-76-5 |
| 2-(4-fluorophenyl)acetaldehyde | 11126322 | 1736-67-0 |
| 1H-inden-1-one | 11815384 | 480-90-0 |
| Naphthalenone | 12446728 | 57392-28-6 |
| 2-fluoro-2-phenylacetaldehyde | 12602096 | 13344-76-8 |
| 8-methylidenebicyclo[4.2.0]octa-1,3,5-trien-7-one | 13167180 | 88180-40-9 |
| 6aH-cyclopropa[a]inden-6-one | 15732192 |  |
| 2-(3-fluorophenyl)acetaldehyde | 15811999 | 75321-89-0 |
| 2-(2-Fluorophenyl)Acetaldehyde | 17770161 | 75321-85-6 |
| 3-fluorohexan-1-ol | 19105682 |  |
| bicyclo[2.2.2]octa-1,3,5-trien-8-one | 19743341 |  |
| 3-oxo-2-phenylprop-2-enal | 21258278 |  |
| 4-(fluoromethyl)benzaldehyde | 21407901 | 64747-66-6 |
| 3-(fluoromethyl)benzaldehyde | 23080897 | 96258-62-7 |
| hydroxy(phenyl)methanolate | 23517413 |  |
| 2-(2-oxoethenyl)benzaldehyde | 45083582 | 89002-82-4 |
| hexa-2,5-dien-1-ol | 53752206 | 28465-64-7 |
| 2,5-Hexadienal | 53799150 | 24058-41-1 |
| 5-fluoroinden-1-one | 55266475 |  |
| 3-methylidene-6-(oxomethylidene)cyclohexa-1,4-diene-1-carbaldehyde | 56633662 |  |
| 2-fluoropent-3-en-1-ol | 57051182 |  |
| fluoro-(4-fluorophenyl)methanol | 57224117 |  |
| (E)-3-Oxo-2-phenylprop-1-en-1-olate | 59895713 |  |
| fluoro-(2-fluorophenyl)methanol | 66718278 |  |
| bicyclo[3.2.2]nona-1(7),5,8-trien-4-one | 67715125 |  |
| [3-(fluoromethyl)phenyl]methanol | 68528076 |  |
| fluoro-(3-fluorophenyl)methanol | 69304374 |  |
| (2,3-difluorophenyl)-fluoromethanol | 70187444 |  |
| Bicyclo[4.1.0]hepta-1,3,5-triene-7-carboxaldehyde | 71332736 | 102073-01-8 |
| 5-fluorohexan-1-ol | 72823953 |  |
| 4-fluoro-3H-indene-1,2-dione | 83069838 |  |
| 3,3-difluoro-2H-inden-1-one | 83669798 |  |
| bicyclo[3.3.1]nona-1,3,5(9)-trien-6-one | 87233327 |  |
| 4-fluorohexan-1-ol | 87401947 |  |
| 4-formylbenzoyl fluoride | 90160302 |  |
| 2-(2,3-difluorophenyl)-2-fluoroacetaldehyde | 90375715 |  |
| 3-fluoro-2,3-dihydroinden-1-one | 91882489 |  |
| naphthalene-1-carbaldehyde | 101170232 |  |
| oxidooxy(phenyl)methanol | 101334094 |  |
| 5-fluoro-2-methylidene-3H-inden-1-one | 101875887 |  |
| (3S)-3-(fluoromethyl)-2,3-dihydroinden-1-one | 102233594 |  |
| 2-(oxomethylidene)indene-1,3-dione | 102578882 |  |
| 2-(2,4-difluorophenyl)-2-fluoroacetaldehyde | 105435719 |  |
| 1-(2-ethenyl-4-fluorophenyl)ethanone | 108327546 |  |
| 2,4-difluoro-2,3-dihydroinden-1-one | 117942772 |  |
| 2-fluoro-3-methyl-2,3-dihydroinden-1-one | 118515426 |  |
| 1H-Inden-1-one | 119092183 | 67864-38-4 |
| 3-fluoro-3-methyl-2H-inden-1-one | 122380797 |  |

**Supplementary Table S3.** Five-fold random split Support Vector Machine performance metrics. TP: true positives, TN: true negatives, FP: false positives, FN: false negatives, %CC: percentage of instances correctly classified, MCC: Matthews correlation coefficient.

| Dataset | TP | TN | FP | FN | %CC | Precision | Recall | MCC |
| --- | --- | --- | --- | --- | --- | --- | --- | --- |
| Training | 8.17±1.12 | 29.83±1.35 | 2.50±0.71 | 1.50±0.71 | 0.90±0.03 | 0.77±0.05 | 0.84±0.08 | 0.77±0.07 |
| Test | 3.00±0.76 | 6.17±0.83 | 0.50±0.71 | 0.33±0.44 | 0.92±0.06 | 0.88±0.16 | 0.91±0.12 | 0.83±0.12 |

The Mathews correlation coefficient (MCC) is obtained as follows:

$$MCC=\frac{TP\times TN-FP\times FN}{\sqrt{\left( TP+FP \right)\left( TP+FN \right)\left( TN+FP \right)\left( TN+FN \right)}}$$

$$Precision=\frac{TP}{TP+FP}$$

$$Recall=\frac{TP}{TP+FN}$$

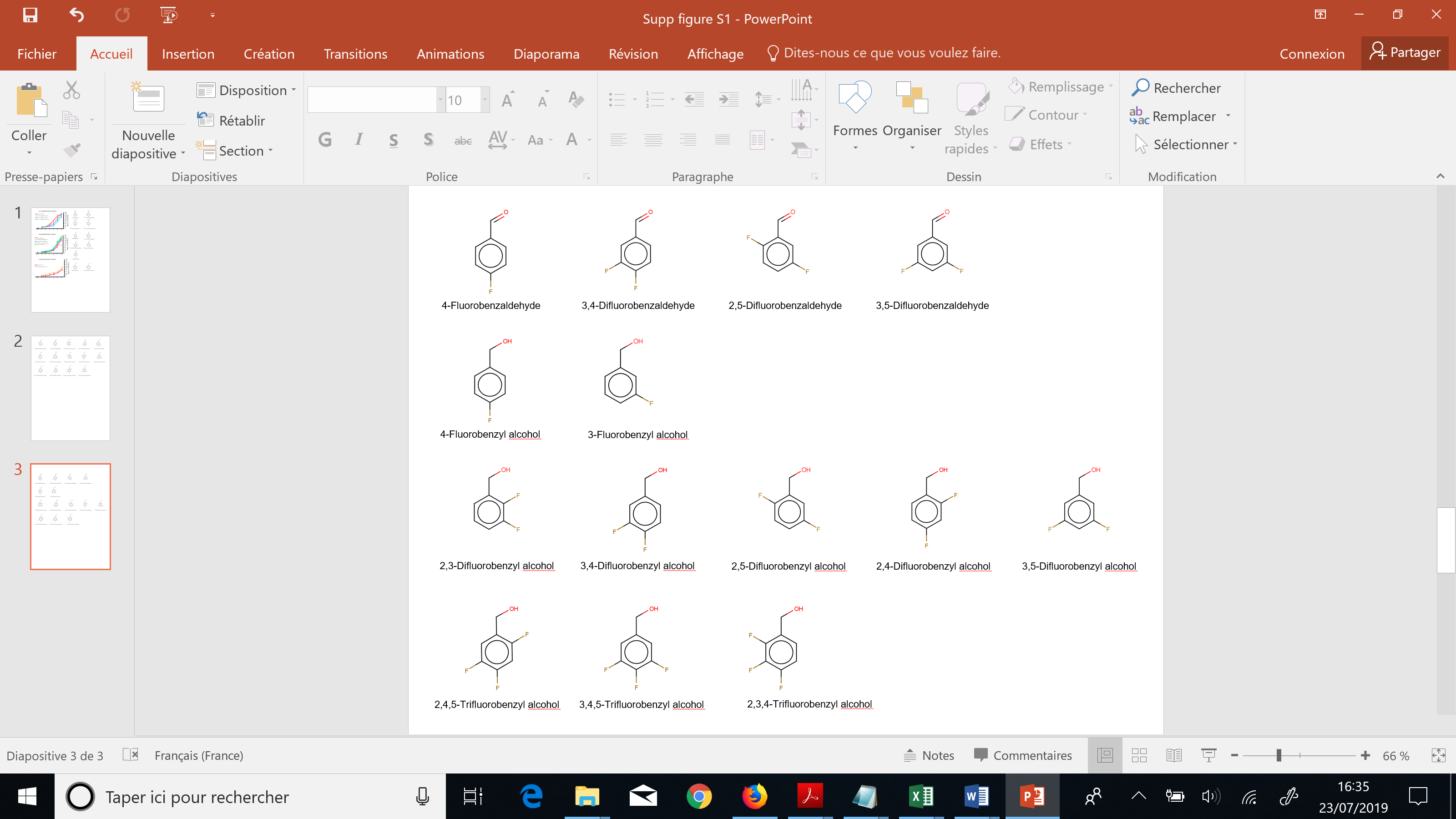


**Supplementary** **Figure S1.**

Chemical structure of predicted but non-active ligands for SlitOR25.


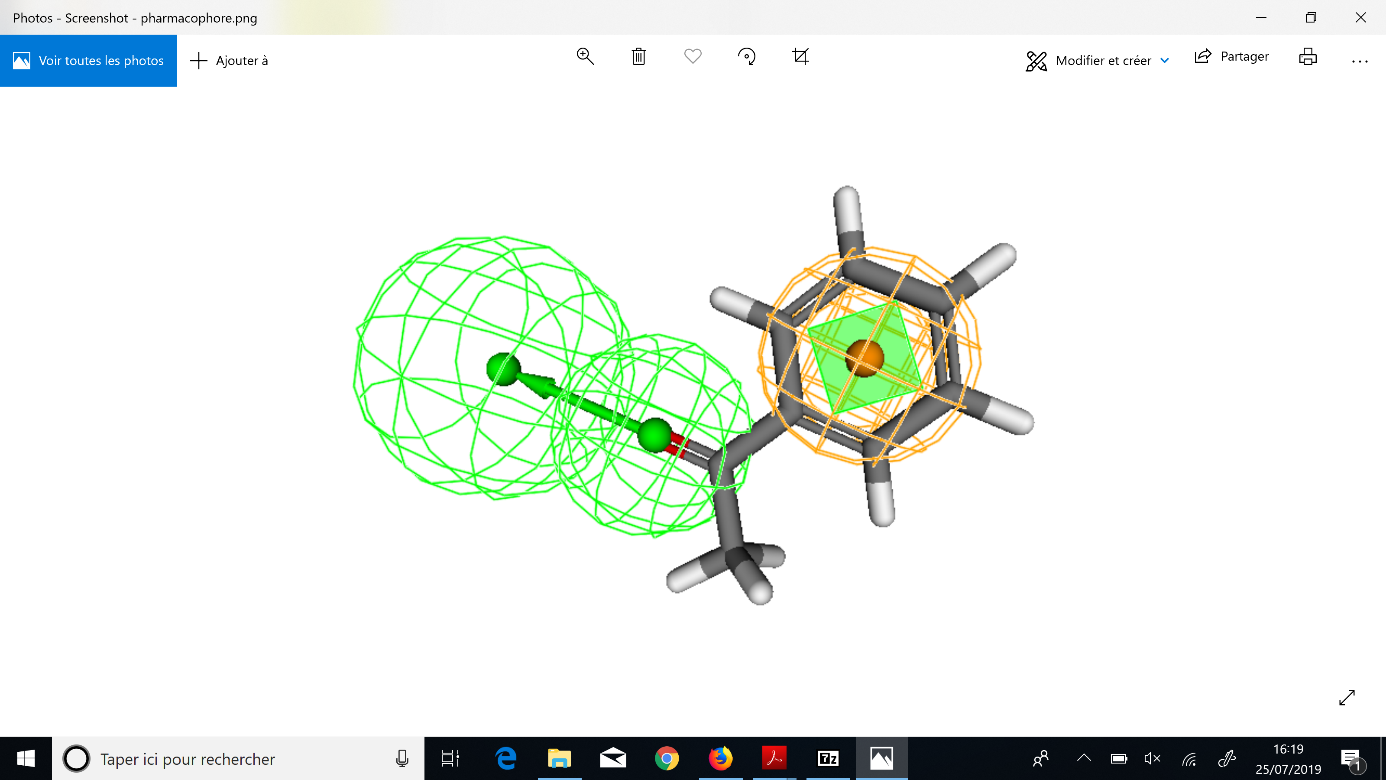


**Supplementary Figure S2. SlitOR25 pharmacophore hypothesis**.

The pharmacophore bears an aromatic cycle (orange sphere) and a hydrogen bond acceptor (green spheres). Acetophenone perfectly fits into this pharmacophore. Note that non-agonists also fit into the pharmacophore model, emphasizing that the model does not accurately discriminate agonists from non-agonists.


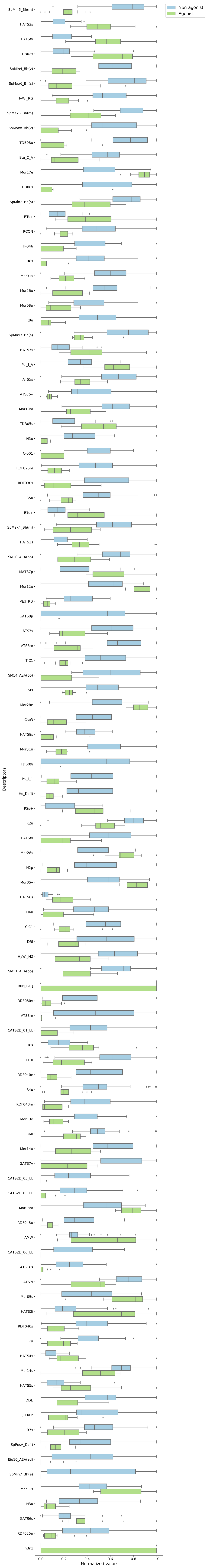

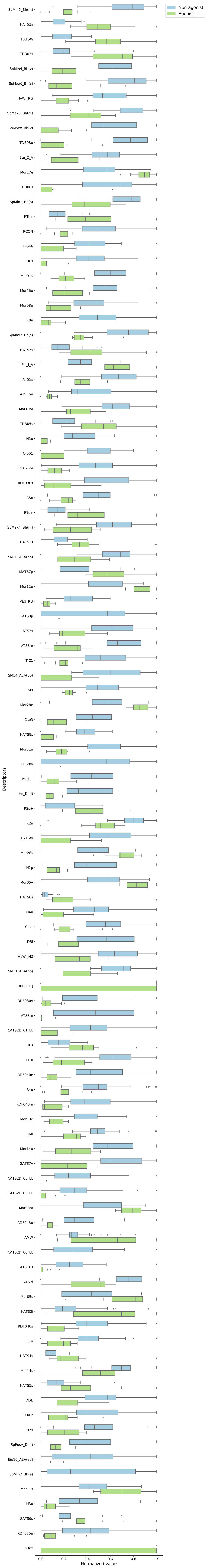

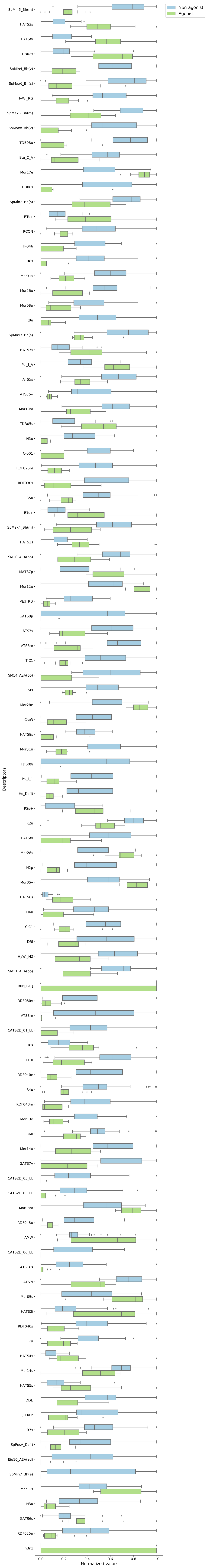

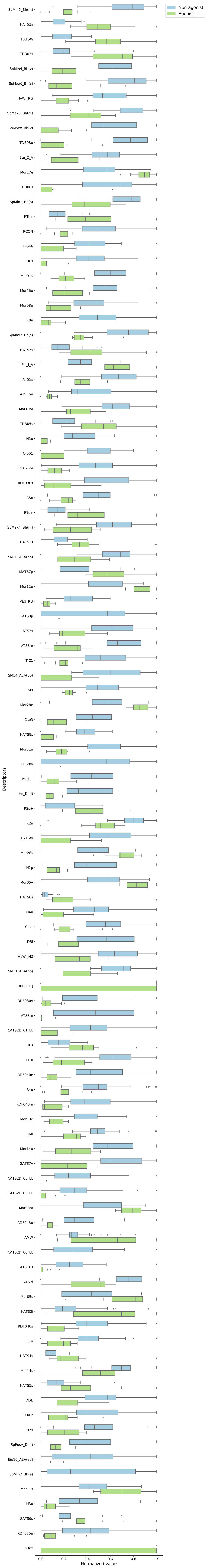


**
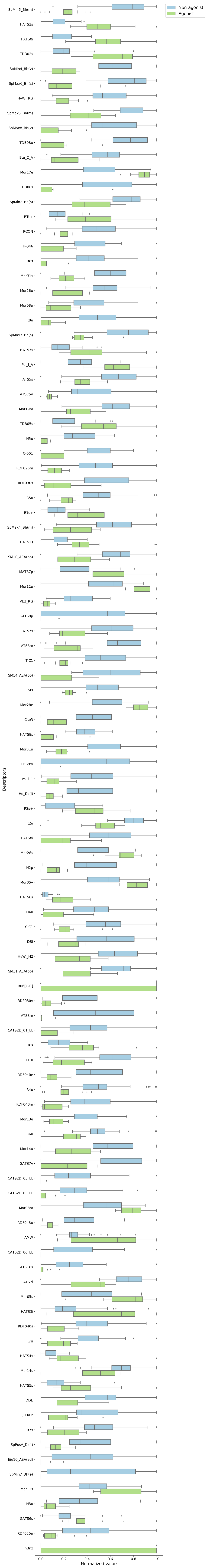

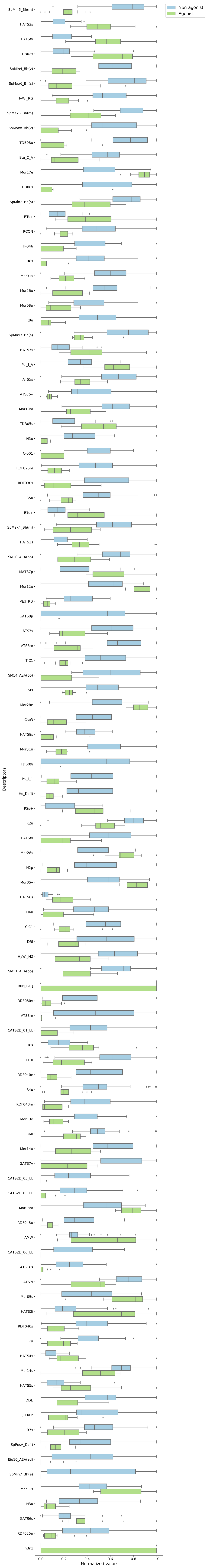

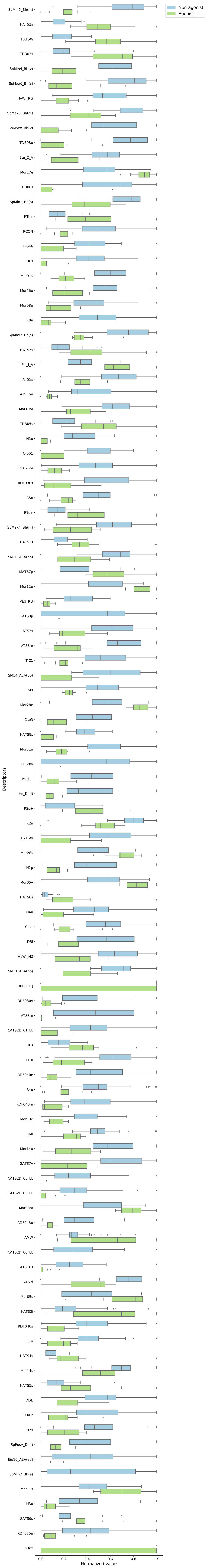

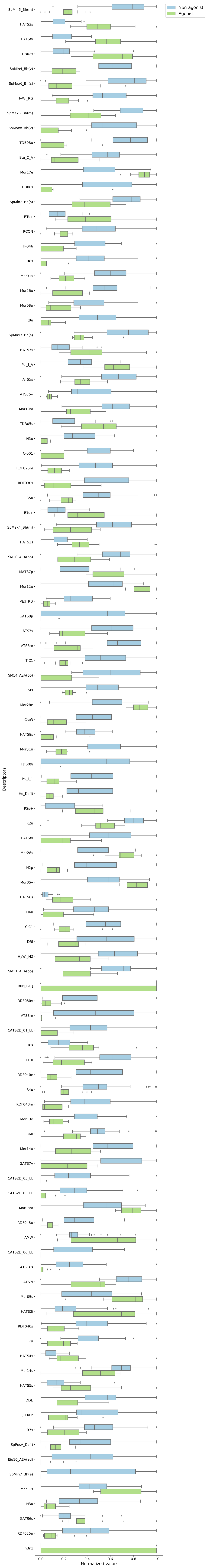
Supplementary Figure S3**.

Descriptors statistically significant (amongst the 394 in total) to discriminate agonists from non-agonists (t-test) are represented as boxplots. The two samples (agonists and non-agonists) were considered as independent. The Benjamini-Hochberg procedure was used to consider the false discovery rate (FDR). Using a FDR of 0.1%, the resulting critical p-value was set to 2.5E-04, which resulted in the identification of the 105 descriptors shown here. Each descriptor values were normalized between 0 and 1 for easier visualization.

**t-SNE calculation:**

The t-SNE implementation of the Python package scikit-learn was used with the following parameters: embedding initialization through principal component analysis (PCA) instead of random, learning rate of 300, early exaggeration of 15, perplexity of 30, and 1000 iterations. Different parameters close to the ones recommended in the package documentation were tested until compounds which are structurally similar were plotted close to each other, and dissimilar molecules were plotted distant from one another.
